# Finite element and preclinical analysis of tissue response to negative pressure wound therapy with a felted foam containing 10 mm through holes

**DOI:** 10.3389/fbioe.2025.1568540

**Published:** 2025-08-21

**Authors:** Amy K. McNulty, Robert P. Wilkes, Brenda Marchand, Shannon Ingram, Samantha Mann, James Sieracki

**Affiliations:** Solventum, St. Paul, MN, United States

**Keywords:** vacuum assisted closure (VAC), debridement, finite element analysis, negative pressure wound therapy with instillation, strain, friction, non-viable tissue

## Abstract

**Introduction:**

Not all wound patients are candidates for surgical debridement. A felted, reticulated open cell foam with an array of 10 mm holes (VFCC) for use with instillation therapy has been used to eliminate non-viable tissue from patient wound beds. The mechanisms for this have not been fully elucidated. The current study elaborates the biomechanical stresses, strains and work imparted to tissue with VFCC versus commonly used reticulated open cell foam (ROCF) dressings.

**Methods:**

Finite element analysis (FEA) measured strain and deformation occurring at the tissue interface with VFCC or ROCF. FEA results were compared to those in a preclinical, porcine sloughy wound model.

**Results:**

The peak maximum principal strain imparted to tissue at –125 mmHg with VFCC was 27.8% versus 0.9% with ROCF. The frictional work around the holes in the VFCC was 0.179 mJ while negligible with ROCF.

**Discussion:**

The FEA model predicted high strains at the sides of the macrodomes of tissue pulled into the through holes and was consistent with slough removal in the preclinical study. Frictional work around the 10mmholes in the VFCC may pin the tissue leading to higher strain energy densities as tissue is pulled into the holes allowing for fracturing and removal of devitalized tissue.

## 1 Introduction

Vacuum Assisted Closure Therapy (VAC Therapy) has been widely used by clinicians and surgeons since its launch in 1995. The most commonly used form of VAC Therapy includes the use of a reticulated, open cell polyurethane foam with a 400–600 µm pore size. The strains imparted to tissue during VAC Therapy using this dressing are believed to facilitate healing through an increase in proliferation (McNulty et al., 2007), perfusion (Erba et al., 2011; Orgill and McNulty, 2022) and through the removal of edema and infectious materials. Introduced in 2011, VAC Therapy with a modified, felted poly-ester based polyurethane open cell foam (VFC) built upon these mechanisms of action. When used in conjunction with instillation therapy (NPWTi-d), this foam was able to distribute the instilled fluids more homogeneously across the wound bed and in pre-clinical models led to increased production of granulation tissue (Lessing et al., 2011). More recently, this same VFC was modified to include 10 mm holes (VFCC). When used in combination with the instillation of topical solutions, this dressing has been reported to provide mechanical movement at the wound surface which may help to disrupt and soften thick exudate and non-viable tissue allowing for its removal (Teot et al., 2017).

The principle of wound bed preparation teaches the management of wounds to create an optimal environment to facilitate healing (Falanga, 2000; Schultz et al., 2003). TIME is an acronym that has been adopted to guide and manage chronic wound bed preparation. The T in TIME stands for tissue and relates to the assessment and debridement of non-viable tissue and foreign material including necrotic tissue, slough, exudate, biofilm and debris (Leaper et al., 2012). Non-viable tissue may be a nidus for infection and is known to delay healing (Leaper et al., 2012; Wolcott et al., 2009). Debridement is the removal of dead (necrotic) or infected tissue from the wound. It is believed that debridement can initiate healing, and that proper wound bed preparation involves the removal of devitalized tissue (Anderson, 2006). The gold standard for debridement is sharps/surgical debridement. It is the most rapid way to clean a wound bed. However, not all wound care practitioners may feel comfortable using a scalpel and not all patients may be good surgical candidates (Steed, 2004). In large or complex wounds, sharps debridement is conducted in the operating room. Many elderly patients may not be candidates for general anesthesia.

In 2019, a consensus document was published which indicated that NPWTi-d with VFCC dressing could be considered for: a) wounds that contain thick exudate where removal using standard NPWT or NPWTi-d with ROCF may be difficult b) wounds that could benefit from wound cleansing if a delay in sharp debridement occurs c) wound cleansing in patients who are not candidates for sharp debridement (Kim et al., 2019).

It is not feasible to empirically measure strains imparted to tissues by medical devices. To assess strains in and around the wound bed in response to medical device therapies such as NPWT, Finite Element Analysis (FEA) has been used by a number of authors. FEA is a numerical method used extensively in engineering design and analysis to predict and study mechanical forces and deformations within materials. FEA of the biomechanics of NPWT in soft tissue macrostrain (Chen et al., 2019; Comellas et al., 2018; Huang et al., 2014; Zeybek et al., 2017; Wilkes et al., 2011) and microstrain (Saxena et al., 2004, Wilkes et al., 2009a, Wilkes et al., 2009b) has determined the physiological levels of strain associated with NPWT and healing.

The mechanisms by which VFCC facilitates the cleaning of the wound bed have not been fully uncovered. The current study attempts to elaborate the biomechanical stresses, strains and work imparted to tissue with VFCC versus the most often used negative pressure wound therapy reticulated open cell foam (ROCF) dressing as well as VFC. Our present work builds upon previously published studies (Wilkes et al., 2009a). FEA was used to measure strain, deformation and frictional work that occurs at the tissue interface with VFCC versus VFC or ROCF. The study then compares the modeled tissue response of VFCC and VFC foams to the actual response observed in an *in vivo* porcine, derived-slough model.

## 2 Methods

### 2.1 Foam physical measurements

#### 2.1.1 Pore density and pore size

Pore density and pore size were calculated from electron micrograph images collected using a Scanning Electron Microscope (Carl Zeiss Nan Technology Systems, Oberkochen, GE). Images were derived without metal coating, using the variable pressure mode. A 5-quadrant backscatter detector (working distance 7–9 mm) was used. The scale bar on the electron micrographs was used to estimate pore size and pores per inch. No image thresholding or figure manipulation was used; rather the pore sizes were simply measured using the scale bar associated with each image within the imaging software. A minimum of 38 pores were measured per dressing type.

#### 2.1.2 Surface energy

A calibrated test kit (Accu Dyne Test, Diversified Enterprises, Claremont, NH) was used for surface energy testing of the hydrophobic foams as per Lessing et al. (2011). A 10–20 µL drop of each dyne solution tested was placed onto a level surface of the foam. If the drop beaded, this was an indication that the surface energy of the dyne solution was higher than the dressing. Spreading of the drop in under 2 s indicated that the surface energy of the dyne solution was less than the foam. Subsequent solutions were tested until the dyne solution was found which spread in approximately 2 s. This represented the surface energy of the dressing. Five areas on each dressing and three dressings per foam type were tested. This meant that a total of 15 measurements per foam type were averaged.

#### 2.1.3 Tensile strength

Foams were cut into dog bone shapes (140 mm × 15 mm thick with 25 mm width × 38 mm length grip dimensions and 13 mm width × 35 mm length gauge dimension). Stress at failure was measured by placing the cut foam samples into an Instron (Instron 5,540 Series, Norwood, MA) with a 500 N load cell (Instron 2,530–416, Norwood, MA) and loaded under tension at a uniform rate of 500.0 ± 0.5 mm/min to failure.

#### 2.1.4 Foam stress-strain data

Foams (ROCF; V.A.C.^®^ GranuFoam™ Dressing; VFC; 3M™ V.A.C. Veraflo Cleanse™ Dressing and VFCC; 3M™ V.A.C. Veraflo Cleanse Choice™ Dressing; Solventum Corporation, Maplewood, MN) were preconditioned for a minimum of 24 h at room temperature prior to testing. Following preconditioning, three 1″ diameter foam samples per foam type were submerged in ¼ -strength Dakin’s Solution (Century Pharmaceuticals, Indianapolis, IN) for 24 h to simulate clinical exposure of the foam to dilute sodium hypochlorite solutions such as Dakin’s during instillation therapy.

**FIGURE 1:**
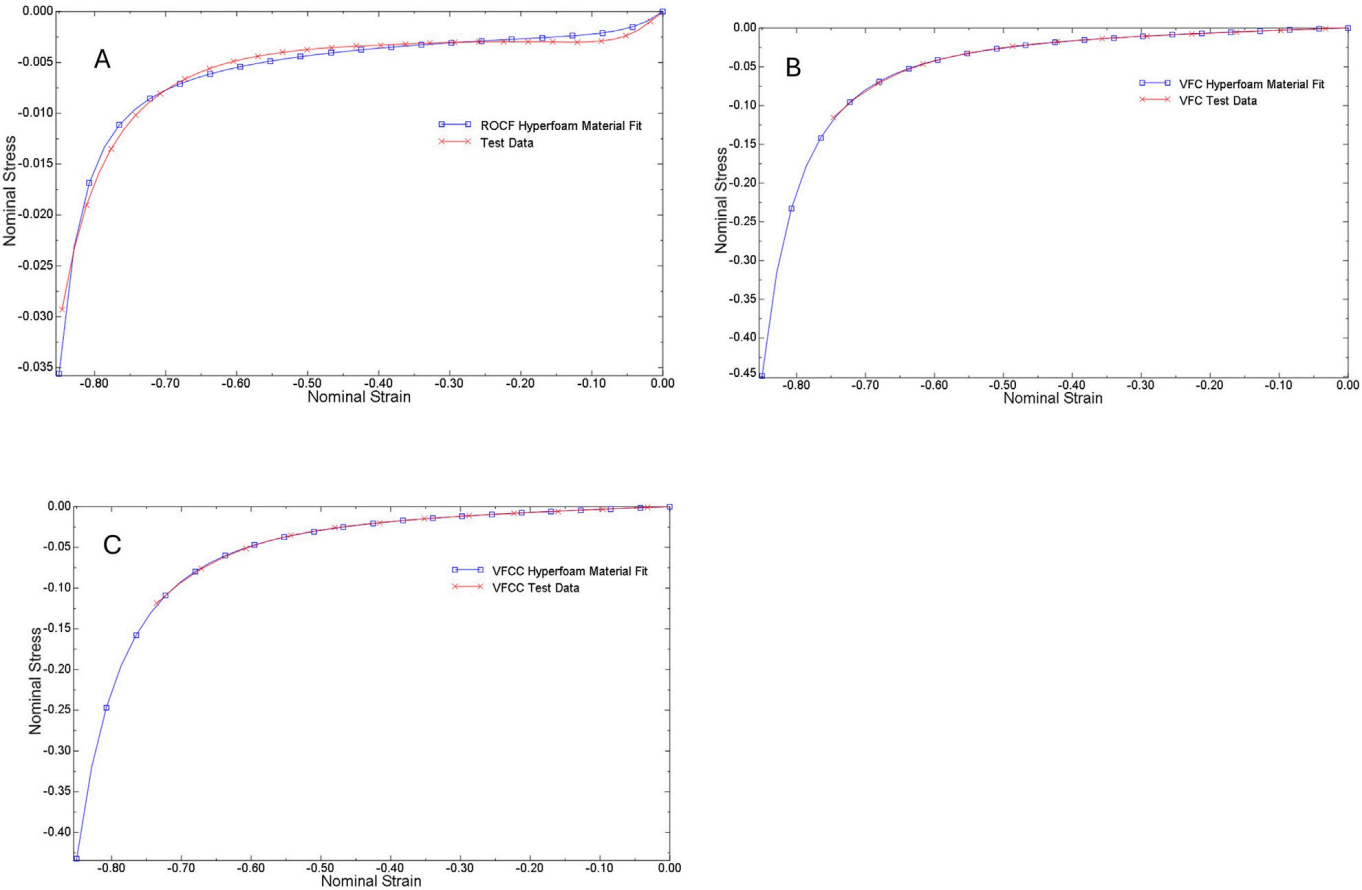
Stress - strain curves for ROCF **(A)**, VFC **(B)** and VFCC **(C)**. Test data from compression testing is shown by the red lines while the Abaqus fit data is shown by the blue lines. Abaqus evaluation of test data generated hyperfoam material coefficients.

Following Dakin’s exposure, foam samples were compressed between two smooth steel platens at a constant rate to 70% compression (30% of the original height) using a TA.XT.plus Texture Analyzer (Stable Micro Systems, Surrey, United Kingdom). The force required to compress the foam samples was recorded and the compression data was converted to stress-strain curves and then evaluated by Abaqus (Figure 1) to generate hyperfoam material coefficients (n = 2) for each foam (Table 1). No lateral expansion of the foams was observed during compression, therefore the foams were assigned a Poisson’s ratio = 0, as is typical for reticulated open cell polyurethane foams (Brincat et al., 2014).

**TABLE 1:** Hyperfoam material parameters obtained by evaluation of uniaxial compression test data using Abaqus. It was observed that the foams did not expand laterally during compression testing, indicating Poisson’s ratio, ν = 0.

Material	*i*	*µ*_*i*_ *(MPa)*	*α*_*i*_	*ν*_*i*_
ROCF	1	2.69 × 10^−2^	25	0
	2	2.02 × 10^−6^	−4.28	0
VFC	1	1.05 × 10^−2^	0.913	0
	2	7.07 × 10^−5^	−4.48	0
VFCC	1	1.21 × 10^−2^	3.22	0
	2	3.46 × 10^−3^	−1.3	0

### 2.2 FEA test method

#### 2.2.1 Construction of the FEA model

**FIGURE 2:**
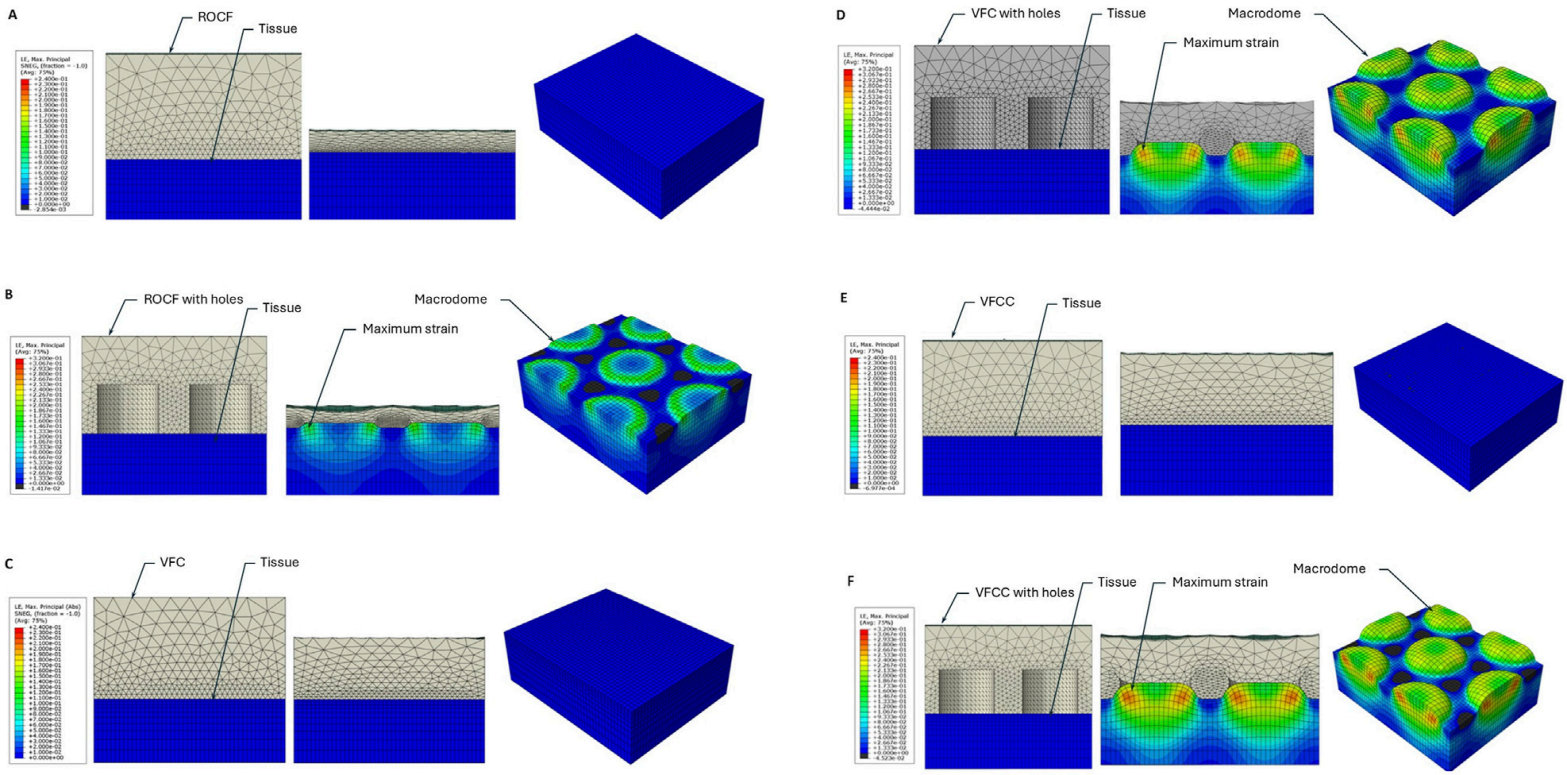
FEA heat map of maximum principal strain for ROCF or VFCC with and without through holes. **(A)** ROCF without 10 mm through holes. **(B)** ROCF with 10 mm through holes. **(C)** VFC without 10 mm through holes. **(D)** VFC with 10 mm through holes. **(E)** VFCC without 10 mm through holes. **(F)** VFCC with 10 mm through holes. In each panel the left figure shows the foam (grey) and tissue (blue) prior to the application of −125 mmHg negative pressure. The middle figure shows the cross-sectional view of the model following application of −125 mmHg negative pressure and the right figure shows the top view of the tissue under −125 mmHg negative pressure with the modeled foam hidden. Note the high levels of strain which occur in the modeled macrodomes of tissue pulled into the through holes of the VFCC dressing. Labels indicate the modeled foam and tissue layers, the macrodomes of modeled tissue and illustrative areas of maximum strain.

Foams were placed on top of the tissue with the 10 mm holes facing down as shown in Figure 2. Drapes were modeled as neo-Hookean with a Young’s Modulus of 10 MPa and a Poisson’s ratio of 0.4. The tissue was modeled as neo-Hookean with a Young’s Modulus of 0.05 MPa and a Poisson’s ratio of 0.15, representing the drained response (Wilkes et al., 2009a) and hyperelastic material parameters of C10 = 0.011 and D1 = 84, with a damping coefficient α = 20,000 to stabilize the contact response. The drained response for the tissue is referred to because the slow loading rate, over several seconds, allows the interstitial fluids to diffuse through the extracellular matrix in the wound. Viscoelasticity is not included because fluid movement will have stalled after a few seconds of loading.

The drape material is a hyperelastic, Ogden (n = 2) model (Wilkes et al., 2011) with material parameters as follows: Ogden, µ_1_ = 0.0147 (MPa), µ_2_ = 5.44 (MPa), α_1_ = 4.3, α_2_ = 1.97, D1 = 0.01 (MPa^-1^) and D2 = 0. Drape thickness = 0.1 mm.

The ROCF, VFC, and VFCC foams were modeled as hyperfoams. Material parameters are provided in Table 1. These foams are highly permeable to fluid flow, even under uniaxial compression. The measured permeability of ROCF under varying degrees of uniaxial compression in water is provided in Table 2. The 60% compression approximates the VFCC foam’s 3X felted density. Negative pressure (−125 mmHg) distribution measurements under uniaxial compressive strains as high as 0.87 demonstrated that the transmission of negative pressure through water exceeded 80% at 148 mm from the central vacuum connection. Considering the high permeability (Table 2) and slow rate of compression of the foams during therapy cycling, the fluid pressure gradient is negligible and thus permeability was not included in the foam material model.

**TABLE 2:** Permeability of ROCF under uniaxial compression. The 60% compression approximates the VFCC foam’s felted density. Negative pressure (125 mmHg) distribution measurements under uniaxial compressive strains as high as 0.87 demonstrated that the transmission of pressure exceeded 80% at 148 mm from the central vacuum connection.

Material	Degree of compression	Modified permeability (m^4^/N-sec)	
		Water (1 mPa-sec)	Exudate (5 mPa-sec)
Polyurethane Foam ∼35 ppi	**0%**	1.6 × 10^−5^	3.2 × 10^−6^
	**30%**	5.5 × 10^−6^	1.1 × 10^−6^
	**60%**	3.8 × 10^−6^	7.6 × 10^−7^
	**90%**	4.3 × 10^−7^	8.6 × 10^−8^

The drape adhesive irreversibly bonds to the foam and therefore the drape-foam contact was modeled as tied. Contact between the tissue and foams was modeled with a friction coefficient of 0.2, a normal linear contact stiffness = 10, and a critical damping fraction = 0.05.

The 3-D FEA model of VFCC or VFC dressing–tissue interaction was constructed using Abaqus version 2019_09_13 finite element analysis software (Dassault Systemes Simulia Corp, Providence, RI, United States). The geometry consisted of a 48 × 45 mm section representing the central region of the foam plus drape in the simulated wound which was 10 mm high and meshed with hexahedral elements (Figure 2). Symmetric boundary conditions were applied around the lateral edges. Simulated negative pressure was applied at −125 mmHg (0.0167 MPa) to the underside of the drape and the top of the tissue as a linear ramp from 0 over 1 s.

#### 2.2.2 Foam geometries

Foam geometries were created in SolidWorks (Dassault Systemes Simulia Corp). The VFC and VFCC Dressings had a geometry of 10 mm diameter holes on a 7.5 mm hexagonal spacing. The 10 mm holes had a height of 8 mm. The foams were modeled as 16 mm thick and meshed with tetrahedral elements. Mesh resolution was determined by balancing computational cost against the 10 mm hole resolution. VFC and VFCC foam was also modeled without the 10 mm holes to facilitate comparison of the effect of 10 mm holes. ROCF was also modeled with 10 mm diameter holes with 7.5 mm hexagonal spacing and 8 mm height to facilitate comparison of the effect of 10 mm holes. The ROCF foam was a total of 16 mm thick. The ROCF was also modeled without the 10 mm diameter holes representing the commercially available foam. The foams were modeled as a single foam piece created using a two-piece foam construct (a perforated layer and non-perforated layer) tied together in the model.

### 2.3 Preclinical studies

#### 2.3.1 Slough construct

The slough construct was designed to mimic the gelatinous consistency and visual appearance of wound slough often seen in clinical cases. The serum protein materials chosen to make the slough were selected due to their biological relevance (Black et al., 2010; Townsend et al., 2024). The slough also contained *Pseudomonas aeruginosa* and *Staphylococcus aureus* which are two of the most common genera found in slough (Verbanic et al., 2020). Cultures of *Pseudomonas aeruginosa* strain 215 and *Staphylococcus aureus* strain 10943 were grown overnight. This mixture was then used to inoculate 25 mm polycarbonate membranes and allowed to grow for 3 days. Each membrane was then placed in phosphate buffered saline, vortexed, sonicated, and then heat deactivated (biofilm was grown at the Center for Biofilm Engineering, Montana State University). This material was combined with porcine plasma (Lampire Biological Labs, Pipersville, PA) and then was used to soak 5 cm diameter collagen pieces cut from sheets of bovine collagen (SkinTemp^®^ II Collagen Sheets, Human BioSciences, Inc., Gaithersburg, MD). After soaking for 5 min, the constructs were implanted into the wounds, covered with gauze and drape for 3 days prior to initiation of NPWTi.

#### 2.3.2 *In vivo* study design

All animal procedures were performed at Surpass, Inc. (Osceola, Wisconsin). This test facility was accredited by the Association for the Assessment and Accreditation of Laboratory Animal Care, International (AAALAC) and registered with the United States Department of Agriculture to conduct research in laboratory animals. The protocol and its amendments were reviewed by the Institutional Animal Care and Use Committee (IACUC) at the AAALAC registered test facility for compliance with regulations prior to study initiation or implementation of amended activities.

Twelve full thickness excisional wounds (5 cm diameter) were created paraspinally in 6 animals (Yorkshire cross swine). Wounds were created on day 0, and the slough implant was placed in the wound bed on day 1. After the placement of the slough implant, wounds were covered with a non-adherent dressing (Adaptic™, Solventum Corporation) for 3 days. On day 4, wounds were dressed with either VFCC or VFC dressings cut to size of the wound. Dressings were attached to an NPWTi device (V.A.C.^®^ Ulta Therapy Unit, Solventum Corporation) using a pressure sensing pad and tubing set (VeraT.R.A.C. Duo™ Tube Set, Solventum Corporation). The therapy setting was 3.5 h of NPWT at −125 mmHg followed by 10 min of instillation using saline. This regime was continued for a total of 7 days of therapy. Dressing changes occurred on days 1, 4, 6 and 8 with study term at day 11. Wound images were taken at dressing changes.

#### 2.3.3 Granulation tissue thickness

Following the 7 days of treatment, a histological analysis on the wound tissue samples was conducted to enable granulation tissue thickness measurements. At necropsy, wounds were excised *en bloc* and fixed in 10% neutral buffered formalin for 72 h before paraffin embedding. The tissues were then sectioned and stained with hematoxylin and eosin (H&E). Slides were scanned and evaluated for granulation tissue thickness by a board-certified veterinary pathologist.

#### 2.3.4 Wound slough measurements

Wound images were captured at all the procedure dates and were utilized to quantify the amount of slough that was present on the wound bed. Two-dimensional wound images were imported into Fiji ImageJ (Wayne Rasband and contributors, National Institutes of Health, United States) and quantification was conducted via color thresholding and pixel analysis.

#### 2.3.5 Statistics

FEA focuses on calculating a specific output (e.g., stress, strain, displacement) for a given set of inputs. While it can be used to incorporate the effects of input uncertainties via Uncertainty Quantification, the core FEA method itself produces a deterministic result and therefore there is no level of error expected.

For the preclinical experiment, statistical analyses were performed using JMP 17.0 software (JMP Statistical Discovery, LLC, Cary, NC). The Shapiro-Wilk goodness of fit test was utilized to assess the data normality. For data that was normally distributed (granulation tissue thickness), analysis of variance (ANOVA) was used. The alpha level was set at 0.05. For non-normally distributed data (percent slough), the nonparametric Wilcoxon method was utilized to test for differences. The alpha level was set at 0.05.

## 3 Results

Physical testing of VFC, VFCC and ROCF shows that there are differences in the properties of the felted (VFC and VFCC) versus non-felted (ROCF) foams (Table 3). VFC and VFCC densify at approximately 50% compression while the ROCF densifies at 80%. Before foams densify (Table 3, E at 5% strain values) the ROCF is stiffer than VFC and VFCC, but once VFC and VFCC densify they are about 7 times stiffer than ROCF. The felting of the VFC and VFCC foams decreases the pore size versus ROCF by over 50% in the felted direction (Table 3). The smaller pore size in the felted direction also means that the density of pores is more than two times higher in VFC and VFCC (Table 3). The VFC and VFCC are less hydrophobic (surface energy greater than 50 dyne/cm) than ROCF (surface energy of 32 dyne/cm) and have a tensile strength approximately 3 times that of ROCF (Table 3). All foams used in this study were able to transmit pressure with negligible pressure drop across the foam surface; transmission of negative pressure in a compressed foam (87% compressive strain) through water exceeded 80% at 148 mm from the central vacuum connection.

**TABLE 3:** Physical parameters associated with ROCF and VFC/VFCC.

Measured parameter	ROCF	VFC/VFCC
Pore Density (pores/inch)	40–50	120–150
Pore Size (um)	400–600	133–200
Surface Energy (dyne/cm)	32	53–56
Tensile Strength (kPa)	≥10	≥33
E at 5% strain (MPa)	0.0523	0.029
E at 50% strain (MPa)	0.0085	0.058

**FIGURE 3:**
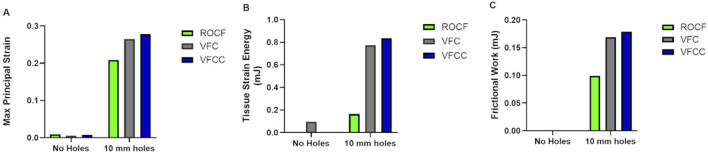
Finite element analysis modeling of Maximum Principal Strain **(A)**, Tissue Strain Energy **(B)** and Frictional Work **(C)** for VFCC, VFC and ROCF with and without 10 mm through holes following application of simulated −125 mmHg negative pressure.

**FIGURE 4:**
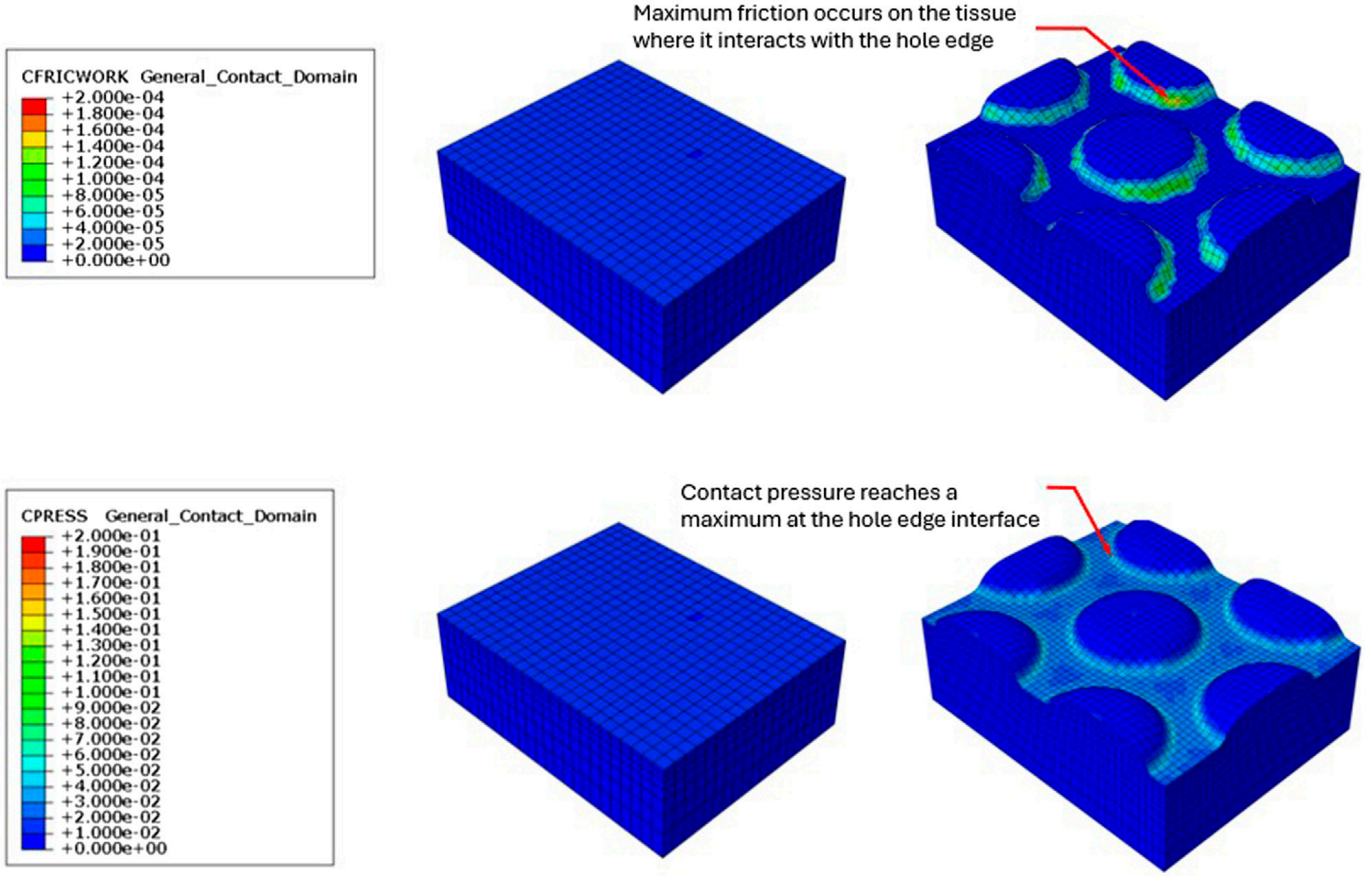
Resulting friction on the surface of the modeled tissue following the application of simulated −125 mmHg negative pressure under VFCC (upper panel). The tissue on the left used VFCC with no through holes as the interface while the tissue on the right used the VFCC with 10 mm through holes as the interface. Note the friction which occurs at the edges of the macrodomes of modeled tissue that have been pulled into the through holes. No friction is evidenced in the modeled tissue on the left when there were no through holes in the VFCC. Contact pressure was maximum at the contact with the hole edges (lower panel).

Figure 2 shows that the maximum principal strain occurs at the edges of the macrodomes which are hereby defined as the domes of tissue pulled into the through holes. The maximum principal strain imparted to tissue at −125 mmHg with VFCC with 10 mm through holes was 27.8% versus 26.5% or 20.9% for VFC and ROCF modeled with 10 mm through holes, respectively. Without the 10 mm through holes, the maximum principal strain imparted to tissue for all foams was less than 1% (see Table 4 and Figures 2, 3). The tissue strain energy associated with the use of VFCC with 10 mm through holes was over 5 times higher than that for ROCF and 1.15 times higher than that for VFC both modeled with 10 mm through holes (Table 4; Figure 3). Without the through holes, the tissue strain energy associated with all foams was 1–3 orders of magnitude lower. Figure 4 shows that measurable friction only is observed in the tissue macrodomes at the periphery of the through holes. The frictional work around the 10 mm holes in the VFCC was 1.8 times higher than for ROCF (0.179 mJ versus 0.099 mJ, respectively; see Table 4 and Figures 3, 4). Frictional work around the 10 mm holes was 1.06 times higher for VFCC than VFC (0.179 mJ versus 0.169 mJ, respectively: see Table 4 and Figures 3, 4). Without the 10 mm through holes, the frictional work associated with the ROCF, VFC and VFCC foams was negligible.

**TABLE 4:** Summary of all FEA data generated for each foam type.

Dressing	Max principal strain	Tissue strain energy (mJ)	Frictional work (mJ)
ROCF – no holes	0.009	0.000137	0.00000
VFC – no holes	0.006	0.095000	0.00000
VFCC – no holes	0.008	0.000136	0.00000
ROCF – 10 mm holes	0.209	0.164551	0.09864
VFC – 10 mm holes	0.265	0.773800	0.16930
VFCC – 10 mm holes	0.278	0.893447	0.17877

**FIGURE 5:**
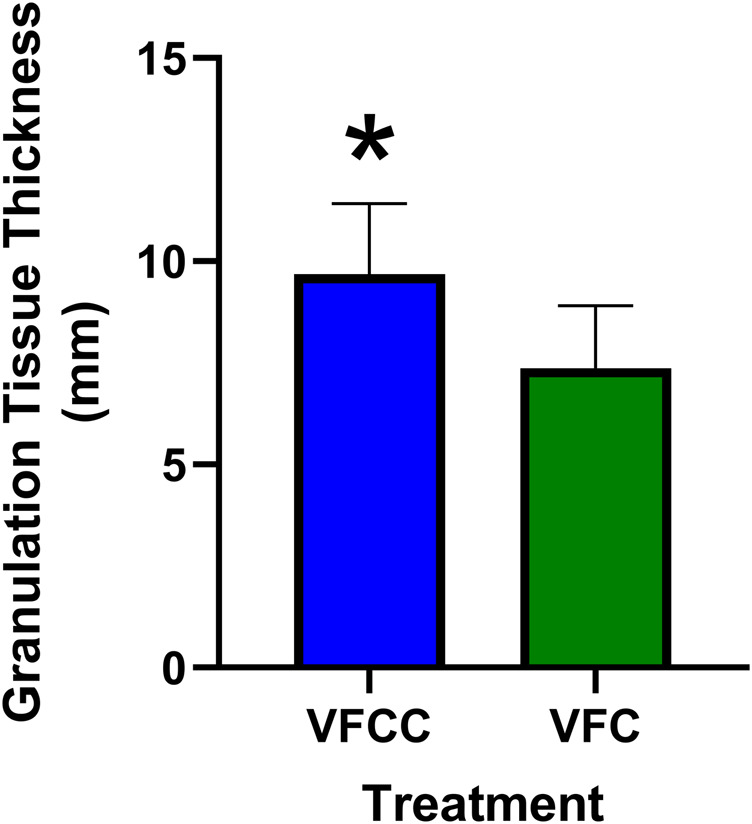
Granulation tissue thickness at study term (day 11) following 7 days of negative pressure wound therapy. At term, wounds were excised *en bloc*, paraffin embedded and stained with hematoxylin and eosin. Slides were evaluated for granulation tissue thickness by a board-certified pathologist. Asterisk indicates that granulation tissue was significantly greater for VFCC (with holes) treated wounds versus VFC (without holes) treated wounds (p = 0.02).

**FIGURE 6:**
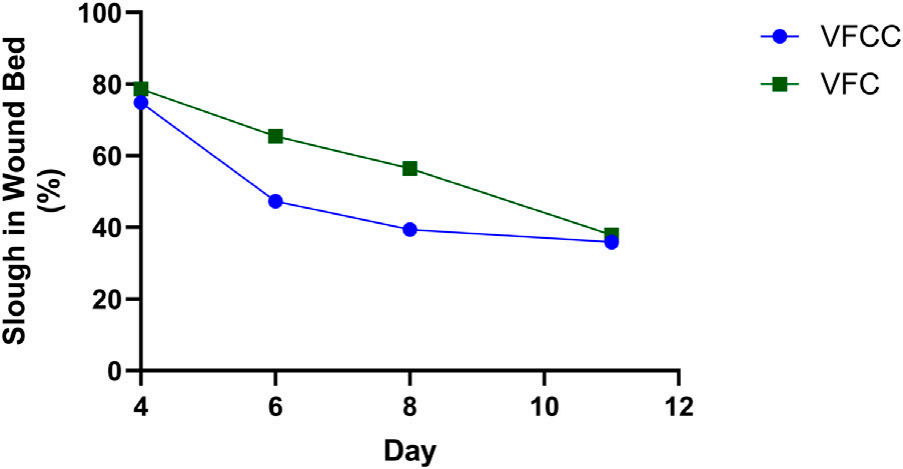
Slough in the wound bed was quantified from wound images captured at each procedure date. There was a decrease in slough over the 7 days of treatment (days 4 through 11) with either VFCC or VFC. Asterisk indicates that there was significantly more slough in the VFC wound at day 6 (=-0.0313). Negative pressure treatments started at day 4 following wound creation (day 0) and slough placement (day 1).

**FIGURE 7:**
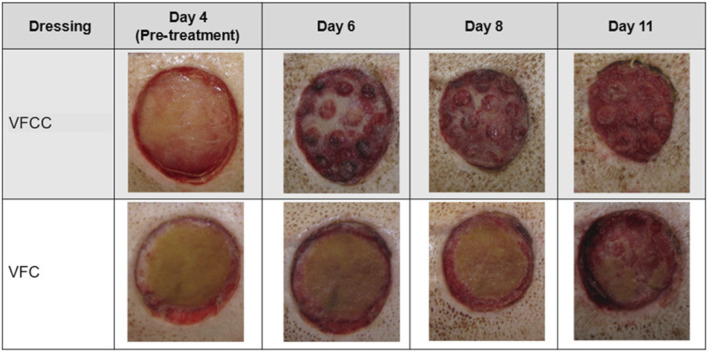
Visual representation of slough removal over the course of the study. Note the difference in appearance between VFCC (with holes) and VFC (without holes) wounds. Especially note the lack of slough on the macrodomes of tissue at day 6. Significantly less slough was present in VFCC wounds at days 6 (p = 0.0313).

The average granulation tissue thickness was significantly greater in wounds treated with VFCC when compared to wounds treated with VFC (9.7 ± 0.6 mm vs. 7.4 ± 0.5 mm, respectively, p = 0.02; Figure 5). Wound sites treated with VFCC had a reduction of slough from 75% on day 4 to 36% by day 11 (after 7 days of treatment). Similarly, wounds treated with VFC had a reduction of slough from 78% on day 4 to 38% by day 11. Figure 6 shows that on day 6, the VFCC wound was covered with significantly less slough at 47% than VFC wounds at 65% (p = 0.0313). On day 8 there was no statistically significant difference between VFCC and VFC slough coverage (40% vs. 56%, respectively; p = 0.0813). There was very little slough observed in the macrodomes of tissue (pulled into the 10 mm through holes of VFCC) at days 6 and 8 versus the layer of slough across much of the VFC treated wounds (Figure 7).

**FIGURE 8:**
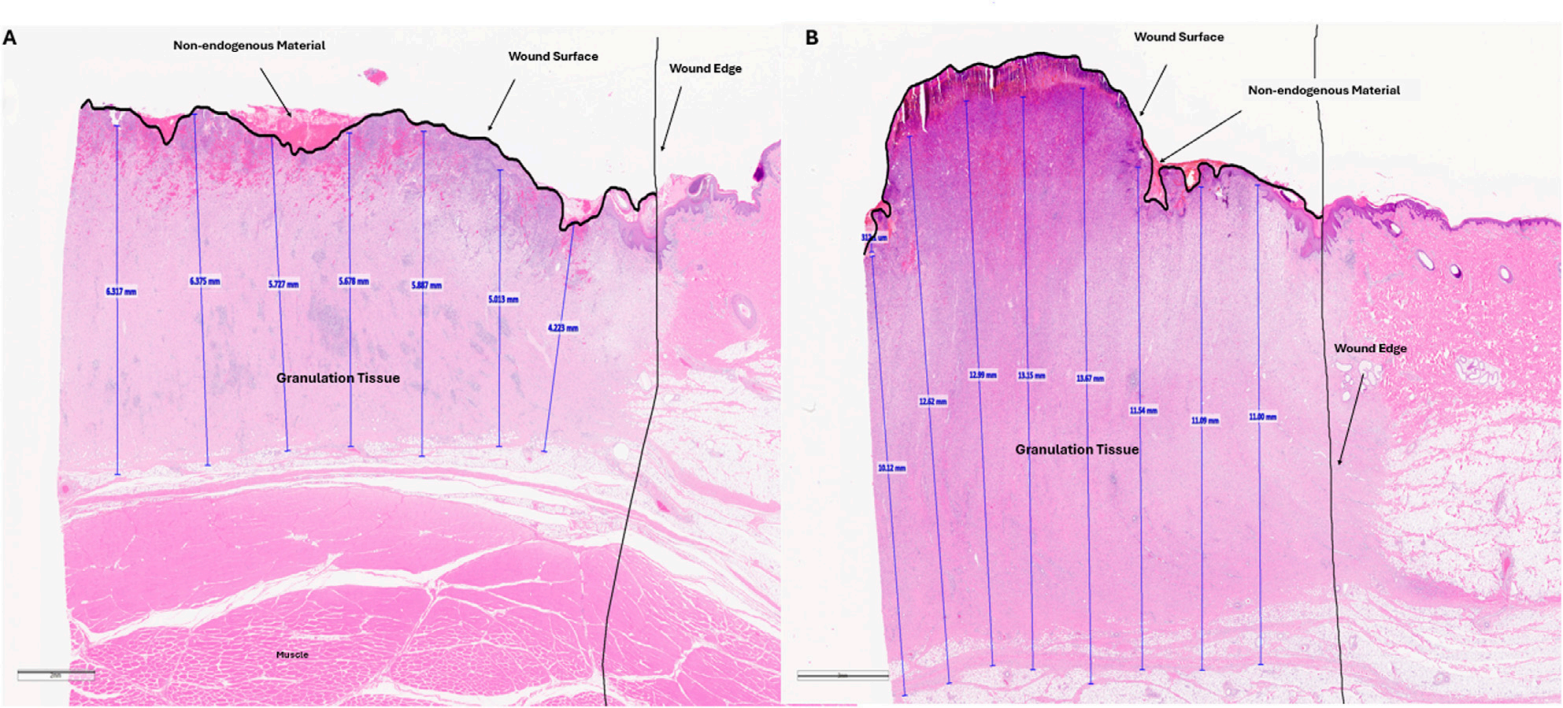
Representative histology images of wound tissue at study term (day 11). Paraffin embedded tissues were sectioned and stained with hematoxylin and eosin. Granulation tissue (vertical blue lines) was measured every 2mm across the wound bed. These values as well as the wound edge and wound surface are annotated by the pathologist. Non-endogenous material above the wound surface, potentially indicative of slough is shown. This material is not intact as shown by cracks in the staining. Panel A is an annotated, representative histology image for a VFC treated wound. Panel B is an annotated, representative histology image for a VFCC treated wound.

## 4 Discussion

The data presented herein show that the through holes in VFCC foam led to higher strains imparted to the tissue than for VFC or ROCF foams with no through holes. Foam stiffness, frictional work and strain imparted to tissue seem to play an important role in the mechanisms of action of the VFCC foam. The higher modulus of VFCC when densified provides sufficient support to allow the soft tissue to rise in the holes without contacting the foam layer above the through holes.

With the specified holes in the foam, frictional work around the 10 mm holes may hold the tissue/slough/devitalized tissue in place, leading to higher (macro) strain energy as the tissue is pulled up into the holes. This frictional work done by the dressing with NPWTi-d may allow for fracturing and removal of the devitalized tissue following softening and solubilization during the instillation phase (Kim et al., 2019). The preclinical results using the VFCC foam demonstrated slough removal and an increased granulation response corresponding to the FEA results. The holes in the VFCC may allow for islands of macrostrain across the wound bed, which in turn assist with the hydromechanical removal of slough and devitalized tissue.

Peak maximum principal strain imparted to tissue at −125 mmHg was 27.8% for VFCC and 0.8% for VFCC modeled without through holes. The frictional work around the holes in the VFCC was 0.18 mJ. When the modeled holes were removed, frictional work across the foam was negligible. The strain energy imparted to tissue with VFCC was approximately 0.89 mJ and 1.36 × 10^−4^ mJ for VFCC modeled without holes. For debridement, frictional work and strain imparted to tissue play an important role. Without the specified holes in the foam, the frictional work and strain imparted to the tissue are minimal.

The stiffer VFCC foam is associated with higher strains imparted to the tissue. Specifically, there is frictional work around the 10 mm holes which may pin the tissue in place under the through holes and therefore allow for higher principal strain and strain energy as the tissue is pulled up into the holes. This work done by the dressing may allow for fracturing of the devitalized tissue which then may be removed during instillation therapy.

The FEA model predicts the highest strain occurs at the edges of the macrodomes of tissue that is pulled up into the 10 mm through holes. This modeling was borne out in the *in vivo* study. Figure 6 shows that on day 6 there appeared to be less slough around the macrodomes of tissue.

Figure 6 showed that there was a greater decrease in the percentage of slough on the wound bed of sites treated with VFCC with holes compared to VFC without holes. In our FEA, the VFCC foam imparted higher values of max principal strain, tissue strain energy and frictional work than VFC (which does not have through holes but has a similar stiffness to VFCC). Wound sites treated with VFCC had a reduction of slough from 75% on day 4 to 36% by day 11 (after 7 days of treatment). Similarly, wounds treated with VFC had a reduction of slough from 78% on day 4 to 38% by day 11. In the intermediate timepoints of the study, the VFCC dressing was able to remove the slough sooner than the VFC dressing (47% compared to 65%, respectively, by day 6).

Slough is a complex, gelatinous biomaterial composed in part from structural, extracellular matrix and blood clot related proteins, leucocytes, and dead and living cells including bacteria and biofilm (Black et al., 2010; Brown, 2013; Percival and Suleman, 2015; Angel, 2019; Townsend et al., 2024). Two of these proteins are fibrin and collagen (Black et al., 2010; Townsend et al., 2024). To date, there is a dearth of information in the literature regarding the biomechanics of slough. Slough is described in the literature as a heterogeneous material, unique to each patient which can be soft, stringy or mucinoid and either loosely or firmly attached to the wound bed (Black et al., 2010; Townsend et al., 2024). Since biomechanical data on slough is not available, the biomechanics of collagen and fibrin were used as surrogates. Studies have shown that the fracture strain of collagen is between 12%–16% while fibrin may be able to stretch 2 to 3- fold prior to fracturing (Litvinov and Weisel, 2017). However, small cracks in fibrin gels have been shown to significantly decrease both the strength of the gel and the rupture strain (Tutwiler et al., 2021). Further, Buehler (2008) showed that the yield strain of uncross-linked collagen was 5%; lower than cross-linked collagen. Shen et al. (2008) indicated that the yield strain of collagen was decreased when collagen fibrils were exposed to repeated or cyclic loading. The 27.8% peak strain for VFCC observed in this study may therefore be enough to rupture the structural proteins in slough. Our leading hypothesis is that the repeated instillation cycles may be able to weaken proteins in the slough in conjunction with the strain imparted to the tissue under the foam to cause ruptures and that the instillation fluid may then infiltrate these ruptures and facilitate the hydromechanical removal of slough during the subsequent negative pressure cycle. The fact that VFCC is able to clean complex wounds of sloughy tissue has been confirmed clinically (Aburn et al., 2021, Blalock, 2019; Chowdhry and Wilhelmi, 2018; Delapena et al., 2020; McElroy, 2019; Obst et al., 2019; Teot et al., 2017). These studies showed that VFCC with cyclic instillation was able to remove or decrease non-viable tissue in a variety of wound types including pressure ulcers (Teot et al., 2017; Obst et al., 2019; Delapena et al., 2020), traumatic wounds (Blalock, 2019), diabetic foot ulcers (McElroy, 2019; Blalock, 2019), burns (Delapena et al., 2020; Teot et al., 2017) and venous leg ulcers (Cole, 2020).

The preclinical study additionally showed that there was significantly greater granulation tissue thickness generated in wounds treated with VFCC (with holes) versus VFC (without holes). This benefit of VFCC with cyclic instillation has also been shown clinically (Fernandez et al., 2017; Matthews et al., 2018; McElroy, 2019; Obst et al., 2019; Teot et al., 2017). It may be that the islands of macrostrain across the wound bed associated with VFCC facilitate granulation tissue production more so than the macrostrain at the edges of the VFC dressing without the through holes. Without the through holes, the friction across the dressing under negative pressure is negligible. Without friction the foam material will slip rather than be pinned when the vertical forces of negative pressure pull the tissue through the holes. This pinning of foam to tissue allows for the generation of the high strain values observed in the macrodomes of tissue. It is hypothesized that in addition to enabling the rupture and removal of sloughy tissue, these high strain values may also facilitate the production of granulation tissue. More work needs to be done to assess the role that the 10 mm holes play in granulation tissue formation.

## 5 Limitations

While this study has revealed important biomechanics associated with the use of NPWTi foams, it is important to discuss some of the limitations in the modeling approach. Firstly, as with all FEA tissue models, homogeneity of the tissue is assumed whereas actual tissue has multiscale variations in these properties. The net result of this is likely that our strains may be over or under predicted. The relative comparison between the foams with and without the 10 mm through holes are still valuable providing important insights. There was no ROCF arm in the preclinical study. The predicted strains were *in silico* calculations. Future studies will add ROCF arms to assess slough removal. The *in silico* tissue was modeled using average physical values from the literature. Future studies could consider varying these parameters to reflect softer or stiffer sloughy tissue.

## 6 Conclusion

FEA modeling of VFCC foam with 10 mm through holes indicates that the maximum principal strain imparted to tissue is 27.8%. This value is above the yield value for collagen reported in the literature. In the preclinical study VFCC Therapy removed more slough following 2 days of therapy than therapy with VFC which does not have the through holes. FEA modeling of VFC without through holes was associated with 2 orders of magnitude less maximum principal strain than VFCC. Clinically, the combination of VFCC with cyclic instillation of fluid has been used to clean complex wounds of sloughy tissue. The strain and friction imparted to the tissue with therapy using VFCC in combination with the potential weakening of collagen due to the cyclic loading of collagen during repeated instillation cycles may be the mechanism of action by which Negative Pressure Wound Therapy with VFCC leads to the hydrodynamic debridement of tissue.

## Data Availability

The raw data supporting the conclusions of this article will be made available by the authors, without undue reservation.

## References

[B1] Anderson I. (2006). Debridement methods in wound care. Nurs. Stand. 20, 65–72. 10.7748/ns2006.02.20.24.65.c4077 16526165

[B2] Angel D. (2019). Slough: what does it mean and how can it be managed. Wound Pract. Res. 27, 4. 10.33235/wpr.27.4.164-167

[B3] Black J. Baharestani M. Black S. Cavazos J. Conner-Kerr T. Edsberg L. (2010). An overview of tissue types in pressure ulcers: a consensus panel recommendation. Ostomy Wound manage. 56, 28–44. 20424291

[B4] Blalock L. (2019). Use of negative pressure wound therapy with instillation and a novel reticulated open -Cell foam dressing with through holes at a level 2 trauma center. Wounds 31, 2. 10.25270/wnds/23081 30485170

[B5] Brincat J. P. Azzopardi K. M. Buttigieg A. Scarpa F. Grima J. N. Gatt R. (2014). Foams as 3D perforated systems: an analysis of their Poisson's ratios under compression. Phys. Status Solidi B 251, 2233–2238. 10.1002/pssb.201484262

[B6] Brown A. (2013). The role of debridement in the healing process. Nurs. Times 109, 16–19. 24358561

[B7] Buehler M. J. (2008). Nanomechanics of collagen fibrils under varying cross-link densities: atomistic and continuum studies. J. Mech. Behave Biomed. Mater. 1, 59–67. 10.1016/j.jmbbm.2007.04.001 19627772

[B8] Chen X. Li J. Li Q. Zhang W. Lei Z. Qin D. (2019). Spatial–temporal changes of mechanical microenvironment in skin wounds during negative pressure wound therapy. ACS Biomaterials Sci. Eng. 5, 1762–1770. 10.1021/acsbiomaterials.8b01554 33405552

[B9] Chowdhry S. A. Wilhelmi B. J. (2018). Comparing negative pressure wound therapy with instillation and conventional dressings for sternal wound reconstructions. Plastic Reconstr. Surg. Glob. Open 7, e2087. 10.1097/GOX.0000000000002087 30859044 PMC6382248

[B10] Cole W. (2020). Early-stage management of complex lower extremity wounds using negative pressure wound therapy with instillation and a reticulated open cell foam with through holes. Wounds 32, 6. 32335516

[B11] Comellas E. Bellomo F. J. Rosales I. del Castillo L. F. Sánchez R. Turon P. (2018). On the feasibility of the computational modelling of the endoluminal vacuum-assisted closure of an oesophageal anastomotic leakage. R. Soc. Open Sci. 5, 171289. 10.1098/rsos.171289 29515846 PMC5830735

[B12] Delapena S. Fernandez L. G. Foster K. N. Matthews M. R. (2020). Negative pressure wound therapy with instillation and dwell time for the management of complex wounds: a case series. Wounds 32, 12. 33561001

[B13] Erba P. Ogawa R. Ackermann M. Avner A. Lino M. Pouya D. (2011). Angiogenesis in wounds treated by microdeformational wound therapy. Ann. Surg. 253, 402–409. 10.1097/SLA.0b013e31820563a8 21217515 PMC3403722

[B14] Falanga V. (2000). Classifications for wound bed preparation and stimulation of chronic wounds. Wound Rep. Regen. 8, 347–352. 11115147

[B15] Fernandez L. Ellman C. Jackson P. (2017). Initial experience using a novel reticulated open cell foam dressing with through holes during negative pressure wound therapy with instillation for management of pressure ulcers. J. Trauma Treat. 6, 5. 10.4172/2167-1222.1000410

[B16] Huang C. Leavitt T. Bayer L. R. Orgill D. P. (2014). Effect of negative pressure wound therapy on wound healing. Curr. Prob Surg. 51, 301–331. 10.1067/j.cpsurg.2014.04.001 24935079

[B17] Kim P. J. Attinger C. E. Constantine T. Crist B. D. Faust E. Hirche C. R. (2019). Negative pressure wound therapy with instillation: international consensus guidelines update. I Wound J. 13, 174–186. 10.1111/iwj.13254 31667978 PMC7003930

[B18] Leaper D. J. Schultz G. Carville K. Fletcher J. Swanson T. Drake R. (2012). Extending the TIME concept: what have we learned in the past 10 years? Int. W J. 9, 1–19. 10.1111/j.1742-481X.2012.01097.x 23145905 PMC7950760

[B19] Lessing C. Slack P. Hong K. Z. Kilpadi D. McNulty A. (2011). Negative pressure wound therapy with controlled saline instillation (NPWTi): dressing properties and granulation response *in vivo* . Wounds 23, 309–319. 25881108

[B20] Litvinov R. Weisel J. W. (2017). Fibrin mechanical properties and their structural origins. Matrix Biol. 60-61, 110–123. 10.1016/j.matbio.2016.08.003 27553509 PMC5318294

[B21] Matthews M. R. Hechtman A. Quan A. N. Foster K. N. Fernandez L. G. (2018). The use of V.A.C. veraflo cleanse CHOICE in the burn population. Cureus 10, e3632. 10.7759/cureus.3632 30705791 PMC6349569

[B22] McElroy E. F. (2019). Use of negative pressure wound therapy with instillation and a reticulated open cell foam dressing with through holes in the acute care setting. Int. Wound J. 16, 781–787. 10.1111/iwj.13097 30784210 PMC7949336

[B23] McNulty A. K. Schmidt M. Feeley T. Kieswetter K. (2007). Effects of negative pressure wound therapy on fibroblast viability, chemotactic signaling, and proliferation in a provisional wound (fibrin) matrix. Wound Rep. Regen. 15, 838–846. 10.1111/j.1524-475X.2007.00287.x 18028132

[B24] Obst M. A. Harrigan J. Wodash A. Bjurstrom S. (2019). Early-stage management of complex wounds using negative pressure wound therapy with instillation and a dressing with through holes. Wounds 31 (PMID), 31184590. 31184590

[B25] Orgill D. P. McNulty A. K. (2022). Theoretical and pre-clinical models of vacuum assisted closure. Surg. Innov. 5, 533–537. 10.1177/15533506221142690 36446390

[B26] Percival S. L. Suleman L. (2015). Slough and biofilm: removal of barriers to wound healing by desloughing. J. Wound Care 24, 498–510. 10.12968/jowc.2015.24.11.498 26551642

[B27] Saxena V. Hwang C. W. Huang S. Eichbaum Q. Ingber D. Orgill D. P. (2004). Vacuum-assisted closure: microdeformations of wounds and cell proliferation. Plast. Reconstr. Surg. 114, 1086–1098. 10.1097/01.prs.0000135330.51408.97 15457017

[B28] Schultz G. Sibbald G. Falanga V. Ayello E. A. Dowsett C. Harding K. (2003). Wound bed preparation: a systematic approach to wound management. Wound Rep. Regen. 11, S1–S28. 10.1046/j.1524-475x.11.s2.1.x 12654015

[B29] Shen Z. L. Dodge M. R. Kahn H. Ballarini R. Eppell S. J. (2008). Stress-strain experiments on individual collagen fibrils. Biophys. J. 95, 3956–3963. 10.1529/biophysj.107.124602 18641067 PMC2553131

[B30] Steed D. L. (2004). Debridement. Am. J. Surg. 187, S71–S74. 10.1016/S0002-9610(03)00307-6 15147995

[B31] Teot L. Boissiere F. Fluieraru S. (2017). Novel foam dressing using negative pressure wound therapy with instillation to remove thick exudate. I Wound J. 14, 842–848. 10.1111/iwj.12719 28244217 PMC7950135

[B32] Townsend E. Cheong J. Z. A. Radzietza M. Fritz B. Malone M. Bjarnsholt T. (2024). What is slough? Defining the proteomic and microbial composition of slough and its implications for wound healing. Wound Rep. Regen. 16, 783–798. 10.1111/wrr.13170 38558438 PMC11442687

[B33] Tutwiler V. Maksudov F. Litvinov R. I. Weisel J. W. Barsegov V. (2021). Strength and deformability of fibrin clots: biomechanics, thermodynamics and mechanisms of rupture. Acta Biomater. 131, 355–369. 10.1016/j.actbio.2021.06.046 34233219 PMC8483248

[B34] Verbanic S. Shen Y. Lee J. Deacon J. M. Chen I. A. (2020). Microbial predictors of healing and short-term effect of debridement on the microbiome of chronic wounds. npj Biofilm Microbiome 6, 21. 10.1038/s41522-020-0130-5 32358500 PMC7195478

[B35] Wilkes R. Zhao Y. Kieswetter K. Haridas B. (2009a). Effects of dressing type on 3D tissue microdeformations during negative pressure wound therapy: a computational study. J. Biomech. Eng. 131, 031012. 10.1115/1.2947358 19154071

[B36] Wilkes R. Zhao Y. Cunningham K. Kieswetter K. Haridas B. (2009b). 3D strain measurement in soft tissue: demonstration of a novel inverse finite element model algorithm on MicroCT images of a tissue phantom exposed to negative pressure wound therapy. J. Mech. Behav. Biomed. Mater. 2, 272–287. 10.1016/j.jmbbm.2008.10.006 19627832

[B37] Wilkes R. Kilpadi D. V. Zhao Y. Kazala R. McNulty A. (2011). Closed incision management with negative pressure wound therapy (CIM): biomechanics. Surg. Innov. 9, 67–75. 10.1177/1553350611414920 21868417

[B38] Wolcott R. D. Kennedy J. P. Dowd S. E. (2009). Regular debridement is the main tool for maintaining a healthy wound bed in most chronic wounds. JOWC 18, 54–56. 10.12968/JOWC.2009.18.2.38743 19418781

[B39] Zeybek B. Li S. Fernandez J. W. Stapley S. Silberschmidt V. V. Liu Y. (2017). Computational modelling of wounded tissue subject to negative pressure wound therapy following trans-femoral amputation. Biomech. Model Mechanobiol. 16, 1819–1832. 10.1007/s10237-017-0921-7 28553679 PMC5671530

