# Supplementary figures and images for: Finite element and preclinical analysis of tissue response to negative pressure wound therapy with a felted foam containing 10 mm through holes
Source: Front Bioeng Biotechnol. 2025 Aug 21;13:1568540. doi: 10.3389/fbioe.2025.1568540 (PMC12409140; doi:10.3389/fbioe.2025.1568540)

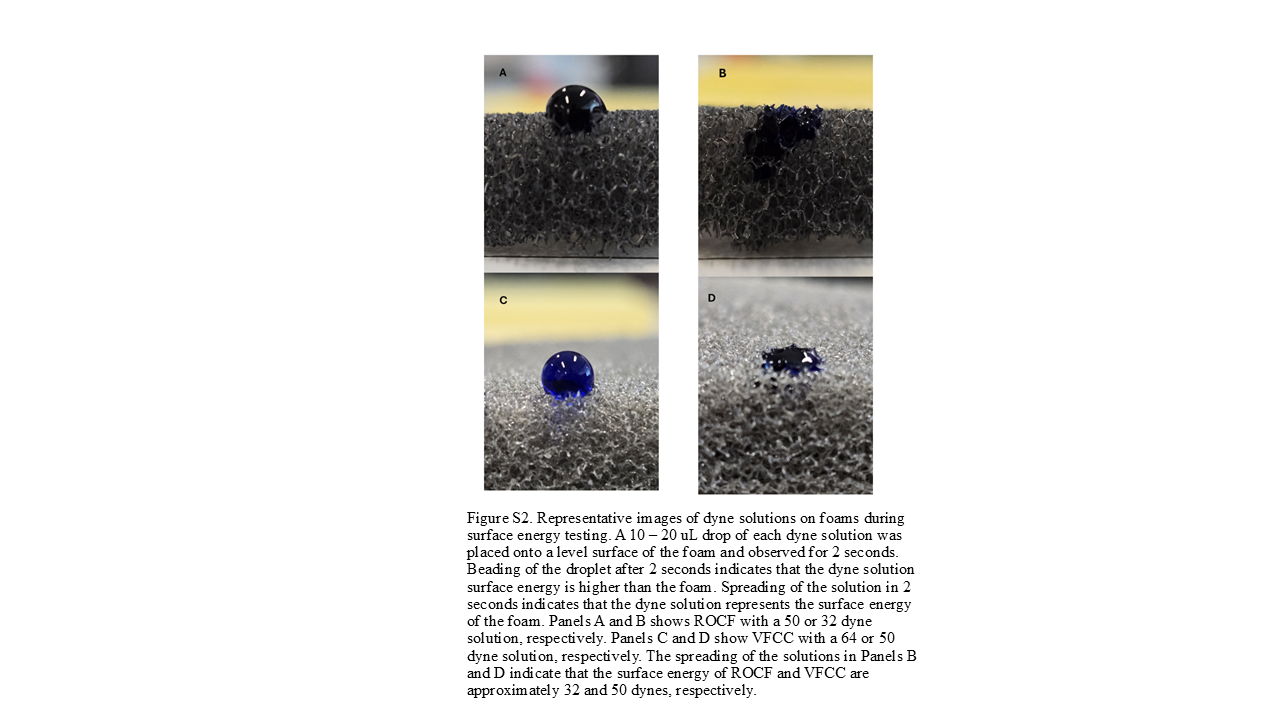

Supplement: Supplementary file 1 [file Image2.tif]

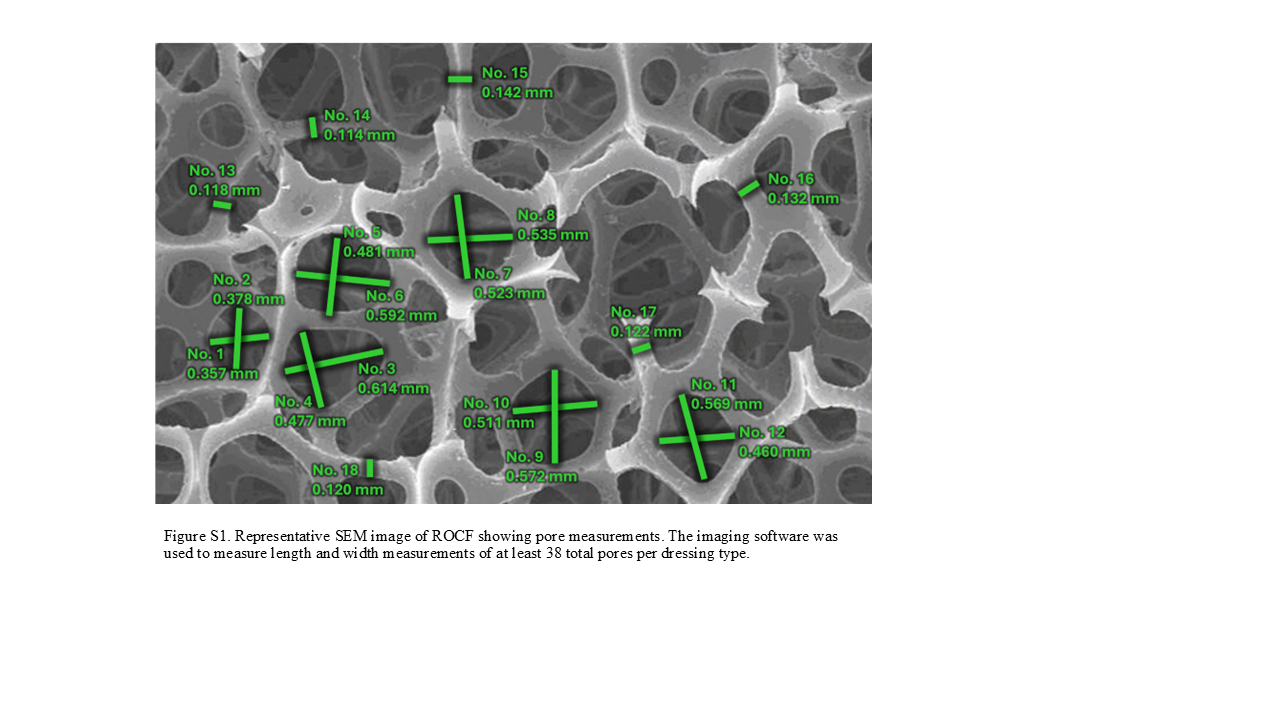

Supplement: Supplementary file 2 [file Image1.tif]
